# Editorial: Coronavirus Disease (COVID-19): Pathophysiology, Epidemiology, Clinical Management and Public Health Response, Volume II

**DOI:** 10.3389/fpubh.2022.913507

**Published:** 2022-06-07

**Authors:** Burc Barin, Zisis Kozlakidis, Fabrizio Ricci, Longxiang Su, Constantinos Tsioutis, Susan C. Welburn, Catherine Ropert, Marco Iosa, Thomas Rawson, Jiufeng Sun, Eugenie R. Lumbers

**Affiliations:** ^1^The Emmes Company, LLC, Rockville, MD, United States; ^2^International Agency for Research on Cancer, World Health Organization, Lyon, France; ^3^Department of Neuroscience, Imaging and Clinical Sciences, G.d'Annunzio University of Chieti-Pescara, Chieti, Italy; ^4^Department of Critical Care Medicine, State Key Laboratory of Complex Severe and Rare Diseases, Peking Union Medical College Hospital, Chinese Academy of Medical Science and Peking Union Medical College, Beijing, China; ^5^School of Medicine, European University Cyprus, Nicosia, Cyprus; ^6^Infection Medicine, Deanery of Biomedical Sciences, College of Medicine and Veterinary Medicine, The University of Edinburgh, Edinburgh, United Kingdom; ^7^Zhejiang University-University of Edinburgh Institute, Zhejiang University School of Medicine, Zhejiang University, Haining, China; ^8^Department of Biochemistry and Immunology, Institute of Biological Sciences (ICB), Federal University of Minas Gerais, Belo Horizonte, Brazil; ^9^Department of Psychology, Sapienza University of Rome, Rome, Italy; ^10^Smart Lab, IRCCS Fondazione Santa Lucia, Rome, Italy; ^11^Medical Research Council (MRC) Centre for Global Infectious Disease Analysis, Jameel Institute, School of Public Health, Imperial College London, London, United Kingdom; ^12^Guangdong Provincial Institute of Public Health, Guangdong Provincial Center for Disease Control and Prevention, Guangzhou, China; ^13^School of Biomedical Sciences and Pharmacy, College of Health, Medicine and Wellbeing, University of Newcastle, Newcastle, NSW, Australia; ^14^Pregnancy and Reproduction Program, Hunter Medical Research Institute, New Lambton Heights, NSW, Australia; ^15^Priority Research Centre for Reproductive Sciences, University of Newcastle, Newcastle, NSW, Australia

**Keywords:** coronavirus-COVID-19, pathophysiology, epidemiology, clinical management, public health response

## Introduction

Since the declaration of the SARS-CoV-2 outbreak a Public Health Emergency of International Concern by the World Health Organization (WHO), 6.2 million associated deaths have been reported and the multi-disciplinary work of researchers worldwide has provided a far deeper understanding of COVID-19 pathogenesis, clinical treatment and outcomes, mortality, dynamics governing disease spread, period of infectivity, and containment interventions. The required rapid processing and spread of accumulated scientific information would not have been possible if not for the special focus and attention given by scientific journals such as Frontiers in Public Health.

Following on from the success of the first Frontiers COVID-19 Research Topic, featuring 400 original research articles (Doolan et al.), Volume II of a dedicated COVID-19 Research Topic was opened for submissions. Relative to Volume I of the Research Topic, which was broad in scope, the follow-up volume, starting in August 2020, focused primarily on areas of public health and medicine, addressing the requirements of the pandemic at the time. Submissions were solicited for the article types of Original Research, Review, Mini-Reviews, Systematic Reviews, Research Protocol, Opinion and Hypothesis, and special emphasis/invitation was promoted to the following topics: (1) Detection, investigation, surveillance, management and control of coronavirus outbreaks; (2) Determination of risk factors and prognostic markers; (3) Molecular and genomic epidemiology investigations of sources and modes of transmission; (4) Clinical trials of anti-infective therapeutics, companion diagnostics or patient care pathways; (5) Public health interventions for prevention, vaccine efficacy and immunization program effectiveness; (6) Natural history of COVID-19 clinical disease spectrum in different populations; (7) Systematic reviews and meta-analysis of COVID-19 epidemiological studies and surveillance data; (8) Pre-clinical development and clinical trials of therapeutic agents for COVID-19; (9) Pre-clinical development and clinical trials of COVID-19 candidate vaccines; (10) Clinical immunology of COVID-19; (11) Long-term sequelae from COVID-19 infections; (12) COVID-19 in pregnancy and potential long term impact on maternal and infant health; (13) Management of post-COVID-19 recovery and rehabilitation; (14) Community, culture, and technology-based interventions; (15) Analysis of social and behavior assumptions underpinning epidemiological models of viral transmission; (16) Understanding of social, economic and political costs of public health interventions such as lockdown, self-isolation and social distancing; (17) Implications of the current pandemic for governance and the social justice agenda worldwide; (18) Analyzing current social change to forecast post-pandemic futures; (19) Innovative delivering of teaching and training in medicine; (20) Post-COVID syndrome.

To provide a backdrop, at the time of the launch of Volume II of the Research Topic, 27 vaccines were undergoing human trials, but no vaccine had yet received authorization at that time, as countries worldwide were continuing to battle case numbers and prepare for resurgences. As of the closing of the Research Topic, nine vaccines had obtained Emergency Use Listing by the WHO, and an even higher number were being administered globally. In total, 385 manuscripts were submitted, 162 (42%) of which were accepted. As of May 2022, the Research Topic achieved ~1,206,000 article views and 142,000 article downloads, with readership distributed across the globe. Frontiers, as the publisher of this Research Topic, made a significant contribution to the timely generation and distribution of peer-reviewed contemporary COVID-19 publications, and combined with Volume I, has produced 562 articles with more than 10,000,000 article views thus far.

Among the four areas covered by this Research Topic, primary focus of the accepted manuscripts was Epidemiology (62), followed by Clinical Management (38), Public Health Response (34), and Pathophysiology (25). The accepted submissions comprised of Original Research (83), Brief Research Report (17), Review (15), Systemic Review (11), Mini Review (3), Perspective (10), Opinion (5), General Commentary (1), Hypothesis and Theory (5), Case Report (4), Community Case Study (1), Study Protocol (3), and Methods (1).

## Epidemiology

Of the articles primarily focused on Epidemiology, a clear area of interest was determination of risk factors and prognostic markers for mortality and/or morbidity. This should not come as a surprise given the extent of pressure observed in healthcare systems globally during the initial waves of the pandemic and the urgent need to identify populations at risk and intervention areas of priority. The goal of many investigations was achieving a better understanding of the individuals with increased risk, and prioritizing their needs during patient triage, ensuring timely medical interventions are implemented, and on a higher level, devise and implement public health interventions to decrease the transmission risk to susceptible populations. While some studies focused on general mortality/morbidity in specific geographical regions (Márquez-González et al.; Martins-Filho et al.), others focused on specific disease groups and more vulnerable patient populations, such as those with diabetes (Xiao Y-F. et al.), asthma and COPD (Pardhan, Wood et al.). Some researchers explored the impact of specific factors of interest such as Vitamin D deficiency (Pardhan, Smith et al.) and smoking (Miyara et al.); others explored the impact(s) of concurrent conditions such as acute kidney injury (Gutiérrez-Abejón et al.) and tuberculosis (Song et al.) on disease outcomes. Huang Y. et al., Bai et al., and Liu Z. et al. attempted to identify biological markers predicting mortality/morbidity.

The second most evaluated epidemiological Research Topic was disease surveillance in order to elucidate the geographical, demographic, health-characteristic, and behavioral distribution of confirmed cases at specific time points (or over time). Accurate disease surveillance is fundamental for governments and health care systems for timely implementation of tightening/relaxing measures as warranted, and for resource and treatment planning relative to available capacity. Healthcare workers are a crucial component of the mentioned resources—studies by Choudhry et al. and Feng et al. focused on disease surveillance among healthcare workers. Predicting the size and duration of future outbreaks and new waves was the ultimate goal; hence, many investigators (Yousefinaghani et al.; Shaharudin et al.; Català et al.; Pérez-Reche et al.; Kuhbandner and Homburg) studied prediction of epidemic curves to help guide implementation of timely public health measures. To complement the picture on symptomatic case identification, Li C. et al., Ambrosis et al., and Hashim et al. focused on investigating asymptomatic rates of infection/disease *via* routine testing and/or modeling. Asymptomatic and pre-symptomatic transmission has introduced a greater degree of challenge and uncertainty for monitoring the spread of infection into new clusters.

One of the challenges in controlling and projecting the course of the pandemic has been re-infection of individuals who have recovered from the disease. In order to obtain a better understanding of the factors leading to re-infection with SARS-CoV-2, Shastri et al., Zhu et al., and Xu et al. studied potential risk factors associated with re-infection, including exposure to a different variant of the virus and potential transmission mechanisms.

For measuring the prevalence of asymptomatic infection and vulnerability to re-infection, understanding the magnitude, variability, breadth, and duration of various types of immune responses is important. Many studies focused on evaluation of more easily (and cost-effectively) measurable humoral immune responses, *via* rapid home tests and/or laboratory tests [Ladage et al. (longitudinal); O'Kelly et al. (healthcare setting); Fujita et al. (healthcare workers); Cerino et al.] while Cremoni et al. analyzed humoral and cellular immune responses concurrently.

During the COVID-19 pandemic, frequent informal comparisons were made between SARS-CoV-2 and other SARS virus and influenza infections as well as between the current and previous pandemics in terms of symptoms experienced, severity of outcome, and general impact on public health. Hence, it was important to conduct formal analyses and reviews to better understand the similarities and differences as implemented by Mann et al., Liu L. et al., Nersesjan et al., and Ledberg.

## Clinical Management

The topic of COVID-19 progression and the associated factors was approached from a clinical management perspective as well. Several studies on progression were in the context of an existing comorbidity or factor, such as hypertension (Mubarik et al.), cancer (Guo et al.; Lin et al.; Barranco et al.), smoking (Xie et al.), amongst others. Three studies generated nomograms to help predict patients' expected disease severity and progression based on patient characteristics, incoming medical status at hospital admission and biochemical/other test results (Tu et al.; Chen et al.; Yu et al.), and there were three other studies evaluating the role(s) of specific biomarkers in disease progression (Billoir et al.; Li L. et al.; Hu et al.) to enable use in clinical management if found relevant. There was also a study evaluating any progression of tinnitus in the context of COVID-19 (Beukes et al.).

Among the articles with a primary focus of clinical management, another main area of interest was the evaluation of various new or concomitant therapies with respect to benefits for COVID-19 progression and management. Treatments evaluated included non-invasive vagus-nerve stimulation (Azabou et al.), sodium copper chlorophyllin (Clark and Taylor-Robinson), corticosteroid use in critically ill patients (Li Y. et al.), spironolactone in patients with liver cirrhosis (Jeon et al.), humified warmed carbon dioxide (El-Betany et al.), cyclosporin A (Devaux, Melenotte et al.), lopinavir/ritonavir and darunavir/cobicistat in hospitalized patients (Castelnuovo et al.), integrated traditional Chinese and Western medicine therapy (Yin et al.) and statins (Fan et al.). The breadth of topics covered demonstrate the multitude of approaches considered, as well as indicating the many clinical specialties involved in those investigations.

Accurate, timely COVID-19 diagnosis and tools used for diagnosis was another research area of interest in clinical management. More than an acceptable threshold of false-positives or false-negatives can cause considerable damage both at individual and public health levels. Xiao A. et al. proposed a triage model for differential diagnosis between COVID-19 and Human Influenza A pneumonia *via* classification and regression tree analysis. Comins-Boo et al. discussed validation of a quick flow cytometry-based assay for acute infection based on CD64 and CD169 expression, which was proposed as a new tool for early diagnosis during the pandemic. Panǎ et al. studied the validity of measuring body temperature *via* non-contact infrared temperature monitors for triaging of possible COVID-19 among oncological and transplant patients. Yin et al. evaluated performance of four antigen rapid tests, one automated antigen dosing, and one molecular point-of-care test vs. gold-standard RT-PCR, while Caixeta et al.  conducted a review summarizing the advantages and limitations of salivary tests for diagnosis of COVID-19.

Pregnant women do not seem to be at higher risk of SARS-CoV-2 infection; however, studies have shown an increased risk of developing severe COVID-19 if they are infected, compared with non-pregnant women of a similar age. Furthermore, COVID-19 during pregnancy has also been associated with an increased likelihood of preterm birth. Hence, pregnant women are considered part of the vulnerable patient population that requires detailed studying. There was one study evaluating impact of cesarean section or vaginal delivery on prevention of possible vertical transmission from a pregnant mother confirmed with COVID-19 to a neonate (Cai et al.). Hashim et al., meanwhile, conducted a systematic review to assess the justification for universal screening of pregnant women for COVID-19 prior to admission to labor.

During the pandemic, it was important to protect healthcare workers, another vulnerable population. Jiang et al. argued that automatic positioning technology applied to relocatable CT can minimize the close contact between technologists and patients and effectively improve the protection of medical staff without sacrificing image quality. Kurotschka et al. explored Italian general practitioners' care experiences and practices during the first wave of the pandemic and whether they were part of an organized emergency response. Vlacha et al. and Schöppenthau et al. explored the rate of infection in healthcare workers and associated factors in prevention of infection.

## Public Health Response

Comparison of public health responses has been vital in the quick adaptation of effective mitigation strategies. Sharma et al. and Basnet et al. shared details of the public health response in Nepal, Dorrucci et al. in Italy, Wang Z. et al. in China, Nam et al. in Vietnam, and Boccia in Europe/UK (during the holidays). Public education and awareness about virus transmission and protective practices were examined by Hossain et al. in construction workers, Qarawi et al. in healthcare workers, Islam et al. for proper PPE-related waste disposal, Sewpaul et al. for complying with social distancing regulations and Iboi et al. in preventing outbreaks. Various studies covered strategic and logistic considerations for COVID-19 vaccines, including vaccine prioritization strategies (Zhang Y. et al.), acceptability (Ali et al.; Gerretsen et al.; Qattan et al.), administration logistics (Litaker et al.), and available emerging vaccines and their comparisons (Blumental and Debré).

Shortly after the beginning of the pandemic, it became clear that some of the individuals recovering from the acute phase of the disease have persisting, relapsing or even new onset symptoms over time. This general condition has been referred to in different contexts by multiple names, including “post COVID-19 condition,” “chronic COVID-19 syndrome,” or “long COVID,” amongst others. The WHO suggested a global clinical case definition *via* Delphi consensus method, and included previously published/available case definitions in its publication as well. It was important to define and track this new condition and several studies focused on characterizing this new condition to understand the public health impact. Although individuals having a more severe version of the disease were more frequently impacted by post COVID-19 syndrome, this condition was observed among the mild cases as well. Chowdhury et al. characterized the symptoms experienced and the changes in the biochemical laboratory test values, recommending that both be examined as part of the routine clinical assessment post-disease. General symptom type, frequency and duration of symptoms were evaluated by Salamanna, Veronesi et al., while more specific symptoms and conditions of interest such as neuropsychiatric symptoms (Alper) and new-onset atherosclerosis (Liu and Zhang) were also explored by others. Mei et al. discussed the general impact of long COVID and its impact on healthcare systems; Kelly et al. generated a study protocol for a scalable rehabilitation pathway addressing the immediate requirements for those recovering from COVID-19 in the community.

It is well-known and demonstrated that the impact of the COVID-19 pandemic has not been limited to physical health. High mortality/morbidity rates observed along with the extended duration of the pandemic with new variants over time have had impact on emotional/mental health as well. Han et al. analyzed gender differences in the severity and psychological impact of COVID-19, while Alper presented a case study of an 18 year-old man with a mildly symptomatic illness that has subsequenlty developed depression and anxiety, disruptive interpersonal conflicts, and impairments in attention and motivation. A separate Frontiers Research Topic was specially devoted to “*Psychological, Behavioral, Interpersonal Effects, and Clinical Implications for Health Systems*”. Overall, emotional/mental health and post COVID syndrome assessments should be part of the routine public health assessments and responses during the pandemic.

## Pathophysiology

Understanding the pathophysiology of COVID-19 is crucial in dealing with the severe forms of the disease, identifying individuals with increased risk, and taking timely action toward development and/or implementation of appropriate treatments. Many studies evaluated the genetic polymorphism and expression playing a role in pathophysiology, with primary focus on ACE2 (Devaux, Pinault et al.; Salamanna, Maglio et al.; Barash et al.; Zhang J. et al.). Hussman, Ruetsch et al., Koblischke et al., Qi et al., and Yang L. et al. explored inflammatory factors, mechanisms and pathways, with association to disease severity. In this research area, there were also studies focusing on disease manifestation in different body systems and organs—pulmonary (Busnelli et al.; Yang K. et al.; Qanadli et al.; Grippo et al.), gut and lung microbiome (Burchill et al.), liver (Wang X. et al.; Lou et al.), central nervous system (Xiang et al.), male reproductive system (He et al.), and skin (Jamshidi et al.). ElAbd et al., Kasozi et al., and Huang C. et al. discussed potentially effective treatments for disease outcomes along with anticipated or observed mechanisms for impact.

## Conclusion

As the COVID-19 pandemic continues, with multiple smaller waves anticipated before gradually becoming endemic, many of the evaluated areas under this Research Topic remain relevant. In particular, epidemiological studies continue to feature strongly in the published scientific literature as understanding of the changing dynamics of COVID-19 remains a public health priority. Despite the development of efficacious vaccines and treatments that made a clear difference in addressing severe forms of the disease, more contagious and/or virulent forms of the virus that are able to evade the immune system persist, and are likely to be of concern in the near future. Therefore, concerted efforts on ongoing and new topics remain a crucial part of the continued fight against COVID-19, with accrued information helping prevent similar pandemics in the future as well.

## Author Contributions

BB compiled the individual article summaries and wrote the first draft. BB, ZK, MI, and TR edited for final revisions. All authors reviewed the draft, generated high-level summaries of the accepted Research Topic articles per their Editorial assignments, and approved the final version for publication.

## Conflict of Interest

BB is employed by the Emmes Company, LLC (Emmes). Emmes did not have any involvement with the editorial in terms of funding or content, or the decision to publish. The remaining authors declare that the research was conducted in the absence of any commercial or financial relationships that could be construed as a potential conflict of interest.

## Publisher's Note

All claims expressed in this article are solely those of the authors and do not necessarily represent those of their affiliated organizations, or those of the publisher, the editors and the reviewers. Any product that may be evaluated in this article, or claim that may be made by its manufacturer, is not guaranteed or endorsed by the publisher.

